# Circulating levels of C-reactive protein, interleukin-6 and tumor necrosis factor-α and risk of colorectal adenomas: a meta-analysis

**DOI:** 10.18632/oncotarget.11853

**Published:** 2016-09-06

**Authors:** Xiaoqian Zhang, Shanglong Liu, Yanbing Zhou

**Affiliations:** ^1^ Department of General Surgery, The Affiliated Hospital of Qingdao University, Qingdao, Shandong 266003, P.R. China

**Keywords:** C-reactive protein, interleukin-6, tumor necrosis factor-α, colorectal adenomas, meta-analysis

## Abstract

Results from publications on inflammatory markers of C-reactive protein (CRP), interleukin-6 (IL-6) and tumor necrosis factor-α (TNF-α) and risk of colorectal adenomas are not consistent. A meta-analysis was conducted to explore the above-mentioned associations. Relevant studies were identified by a search of Embase, Medline and PubMed through February 2016. A random effect model was adopted to combine study-specific odds ratio (OR) and 95% confidence interval (95% CI). Between-study heterogeneity and publications bias were assessed. Dose–response relationships were assessed by restricted cubic splines. Nineteen observational studies were included. For highest vs. lowest levels, results from this meta-analysis did not support an association between circulating levels of CRP [OR (95% CI): 1.15 (0.94-1.40)], IL-6 [1.17 (0.94-1.46)] and TNF-α [0.99 (0.75-1.31)] and risk of colorectal adenomas, respectively. The findings were supported by sensitivity analysis and subgroup analysis. In dose-response analysis, the risk of colorectal adenomas increased by 2% [1.02 (0.97-1.08)] for each 1 mg/L increment in circulation CRP levels, 9% [1.09 (0.91-1.31)] for each 1 ng/L increment in circulation IL-6 levels, and 6% [1.06 (0.93-1.21)] for each 1 pg/mL increment in circulation TNF-α levels. Moderate between-study heterogeneity was found. No evidence of publication bias was found. Circulation levels of CRP, IL-6 and TNF-α might be not useful biomarkers for identifying colorectal adenomas, respectively.

## INTRODUCTION

Colorectal cancer is the third most commonly diagnosed cancer in males and the second in females worldwide [1]. In China, colorectal cancer is the fifth most common cancer in males and the fourth in females, and the incidence rate has been increasing since 2000 [2]. In addition to maintaining a healthy body weight, being physically active, minimizing consumption of red and processed meat and alcohol and avoidance of smoking, colonoscopic removal of adenomas could prevent incidence of colorectal cancer and also prevent death from colorectal cancer [1, 3]. Therefore, explore possible biomarkers to identify individuals who would benefit most from screening such as colonoscopy is needed. Adipose tissue, particularly visceral adipose tissue, is recognized as a key regulator of systemic inflammation [4] and can produce a variety of proteins, hormones and cytokines that are collectively defined as adipokines including interleukin-6 (IL-6) and tumor necrosis factor-α (TNF-α) [5–6], and the risk of colorectal adenomas increased by 13% for each 25 cm^2^ increase in visceral adipose tissue area [7]. C-reactive protein (CRP), one of the acute-phase proteins in inflammation, is produced in response to increased circulating IL-6 and TNF-α, etc. [5] Although results from experimental studies indicated that CRP, IL-6 and TNF-α may be involved in the development of colorectal neoplasia [5–6], published data on circulating CRP, IL-6 and TNF-α and the risk of colorectal adenomas are not conclusive [8–37], respectively. Considering no meta-analysis is available to systematically explore the above-mentioned associations, we conducted a meta-analysis of observational studies following the PRISMA statement (Appendix 1) to assess the association between circulating levels of CRP, IL-6 and TNF-α and the risk of colorectal adenomas.

## RESULTS

### Literature search and study characteristics

The flow chart for study inclusion is shown in Appendix 2. A total of 113 articles were reviewed in full text after reviewing titles and/or abstracts. Another 94 articles were further excluded for other reasons (Appendix 2). Finally, a total of 19 studies were included for quantitatively analysis. 11 articles (6,300 cases and 8,622 controls) were included on CRP and risk of colorectal adenomas, and there were 8 articles for IL-6 (2,062 cases and 3,651 controls), and 8 articles for TNF-α (1,796 cases and 2,980 controls). All included studies were case-control or nested case-control studies, and most of studies were conducted in USA and Asia (detailed information are shown in Appendix 3). Colorectal adenoma cases and control subjects were selected by sigmoidoscopy or colonoscopy. The included studies met the quality score of 4–8 stars.

### Quantitative synthesis

#### CRP and colorectal adenomas (Table 1)

**Figure 1:**
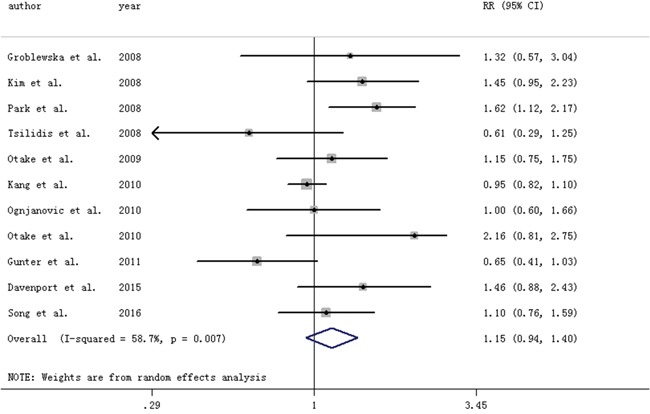
Forest plot for circulating levels of C-reactive protein and risk of colorectal adenomas The size of gray box is positively proportional to the weight assigned to each study, and horizontal lines represent the 95%confidence intervals.

Highest vs. lowest category of circulating CRP levels conferred an OR (95% CI) of 1.15 (0.94-1.40). Moderate between-study heterogeneity was found (I2=58.7%) (Figure 1). Study design (matched case-control study, unmatched case-control study) (P=0.02) and study quality (P=0.02) were found to contribute to the heterogeneity, and a positive association was found in unmatched case-control studies and studies with a relatively low quality, respectively.

**Table 1:** Pooled results on CRP, IL-6 and TNF-α with risk of colorectal adenomas

	CRP						IL-6						TNF-α					
	N	N_case_	N_control_	OR (95% CI)	*I^2^* (%)	*P*	N	N_case_	N_control_	OR (95% CI)	*I^2^* (%)	*P*	N^d^	N_case_	N_control_	OR (95% CI)	*I^2^* (%)	*P*
Overall	11	6300	8622	1.15 (0.94-1.40)	58.7		8	2062	3651	1.17 (0.94-1.46)	40.3		9	1796	2980	0.99 (0.75-1.31)	65.6	
Design						**0.02**						0.59						0.08
MCC	6	3345	3790	0.95 (0.79-1.16)	0.00		3	1146	1514	1.11 (0.72-1.69)	54.5		3	840	769	0.73 (0.50-1.06)	44.9	
CC	5	2955	4832	1.49 (1.22-1.83)	32.5		5	916	2137	1.23 (0.94-1.62)	39.6		6	956	2211	1.19 (0.83-1.70)	64.8	
N_case_						0.59						0.37						0.98
≥300	6	5570	7122	1.10 (0.87-1.40)	65.2		2	1158	1807	0.99 (0.77-1.27)	0.00		2	924	1530	0.98 (0.79-1.22)	0.00	
<300	5	730	1500	1.23 (0.85-1.80)	50.0		6	904	1844	1.30 (0.96-1.76)	47.6		7	872	1450	1.00 (0.65-1.55)	74.0	
Blood sample						0.37						0.96						0.96
Serum	5	3769	5597	1.05 (0.77-1.41)	67.8		5	925	1939	1.12 (0.86-1.47)	16.2		5	638	1290	0.98 (0.57-1.74)	71.8	
Plasma	6	2531	3025	1.26 (0.98-1.63)	39.1		3	1137	1712	1.20 (0.80-1.80)	70.1		4	1158	1690	1.02 (0.73-1.42)	66.4	
Assay method						0.15						0.87						0.92
Immunoturbidimetry/ELISA^b^	6	3741	5197	1.32 (1.06-1.63)	22.5		7	1965	3554	1.17 (0.92-1.48)	---		3	442	989	1.00 (0.56-1.78)	76.9	
Others	5	2559	3125	0.99 (0.74-1.32)	61.6		1	97	97	1.35 (0.58-3.17)	48.2		6	1354	1991	0.96 (0.68-1.36)	62.4	
Country^c^						0.46						0.52						**0.01**
USA	6	2465	3299	1.03 (0.77-1.36)	51.0		6	1906	3398	1.11 (0.88-1.39)	42.5		5	915	2171	1.31 (0.92-1.85)	61.8	
Asia	4	3797	5288	1.32 (0.92-1.90)	78.1		1	118	218	2.06 (1.02-4.16)	---		4	881	881	0.71 (0.51-0.98)	33.0	
Mean age						0.51						0.23						0.34
>55	6	4120	5930	1.23 (0.89-1.68)	66.0		3	1125	1393	0.96 (0.73-1.27)	0.00		6	1015	975	0.88 (0.56-1.38)	67.8	
≤55	5	2180	2692	1.05 (0.84-1.31)	33.1		5	937	2258	1.31 (0.97-1.79)	53.9		3	781	2005	1.16 (0.87-1.56)	50.6	
Male percentage (%)						0.83						1.00						0.63
>55	7	5131	6930	1.17 (0.90-1.52)	69.6		2	389	757	1.28 (0.55-2.97)	75.5		4	663	623	0.94 (0.45-1.98)	77.9	
<55	4	1169	1692	1.13 (0.82-1.54)	28.4		6	1673	2894	1.17 (0.93-1.47)	34.6		5	1133	2357	1.05 (0.79-1.38)	54.0	
BMI^a^						0.36						0.65						**0.04**
>26 kg/m^2^	5	1708	2542	1.00 (0.69-1.44)	60.4		4	1052	2544	1.13 (0.83-1.54)	62.3		4	818	2074	1.36 (0.90-2.06)	71.3	
<26 kg/m^2^	5	4554	6045	1.26 (0.95-1.66)	70.9		3	972	1072	1.29 (0.80-2.08)	44.3		5	978	906	0.76 (0.56-1.01)	28.3	
Score						**0.02**						0.85						0.97
7-8	8	4233	5056	1.02 (0.86-1.22)	38.1		5	1789	3195	1.21 (0.88-1.66)	63.2		4	1421	2416	1.01 (0.74-1.39)	66.4	
4-6	3	2067	3566	1.68 (1.28-2.21)	0.00		3	235	421	1.08 (0.80-1.47)	0.00		5	375	564	0.99 (0.55-1.78)	71.8	

N: number of studies, P: P value from meta-regression with a permutation of 1000 test.

a BMI values were not reported in 1 study.

b Immunoturbidimetry for CRP, and ELISA for IL-6 and TNF-α

c there was 1 study conducted in Poland.

d number of studies including gender-specific results

#### IL-6 and colorectal adenomas (Table 1)

**Figure 2:**
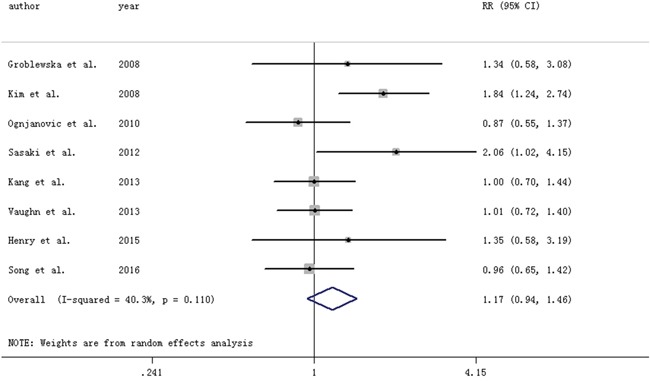
Forest plot for circulating levels of interleukin-6 and risk of colorectal adenomas The size of gray box is positively proportional to the weight assigned to each study, and horizontal lines represent the 95%confidence intervals.

Highest vs. lowest category of circulating TNF-α levels conferred an OR (95% CI) of 1.17 (0.94-1.46). Low between-study heterogeneity was found (I2=40.3%) (Figure 2). No variable was found to contribute to the heterogeneity.

#### TNF-α and colorectal adenomas (Table 1)

**Figure 3:**
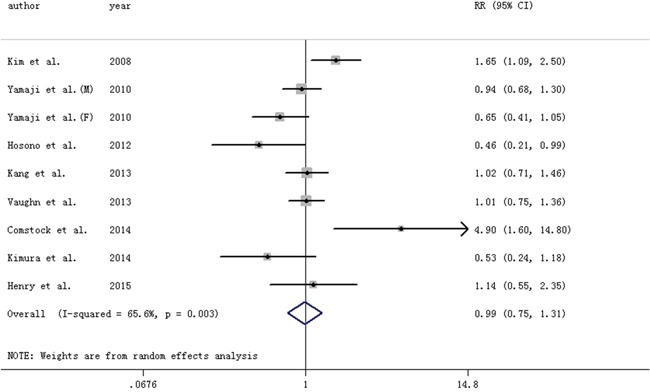
Forest plot for circulating levels of tumor necrosis factor-α and risk of colorectal adenomas The size of gray box is positively proportional to the weight assigned to each study, and horizontal lines represent the 95%confidence intervals. M: male, F: female.

Highest vs. lowest category of circulating TNF-α levels conferred an OR (95% CI) of 0.99 (0.75-1.31). Moderate between-study heterogeneity was found (I2=65.6%) (Figure 3). Country where the study was conducted (P=0.01) and BMI (P=0.04) may contribute to the heterogeneity, and an inverse association was found for studies conducted in Asia.

### Publication bias and sensitivity analysis

There is no evidence of publication bias in the analysis of CRP (P=0.33), IL-6 (P=0.34) and TNF-α (P=0.96) and risk of colorectal adenomas, respectively. No individual study had an excessive influence to the pooled effect in sensitivity analysis in the above-mentioned analysis. In addition, similar results were found after excluding the ORs calculated from mean and standard deviation [CRP: 1.18 (0.93-1.50), 2 studies [21, 26] excluded; IL-6: 1.17 (0.92-1.48), 1 study [26] excluded; TNF-α: 1.11 (0.84-1.47), 2 studies [11, 16] excluded].

### Dose-response analysis (Appendix 4)

The departure from a linear relationship was not significant between CRP (P_for nonlinearity_=0.18), IL-6 (P_for nonlinearity_=0.35) and TNF-α (P_for nonlinearity_=0.87) and risk of colorectal adenoma, respectively. The risk of colorectal adenomas increased by 2% [1.02 (0.97-1.08)] for each 1 mg/L increment in circulation CRP levels (eight studies [8, 10, 17, 20, 22–25] including 4,981 cases), 9% [1.09 (0.91-1.31)] for each 1 ng/L increment in circulation IL-6 levels (five studies [8, 13, 15, 20, 25] including 1,763 cases), and 6% [1.06 (0.93-1.21)] for each 1 pg/mL increment in circulation TNF-α levels (four studies [12–13, 18, 25] including 1,452 cases), respectively.

## DISCUSSION

Results from this meta-analysis suggested that circulating levels of CRP, IL-6 and TNF-α were not associated with the risk of colorectal adenomas. The findings were consistent in sensitivity analysis and dose-response analysis. Moderate between-study heterogeneity was found. No evidence of publication bias was found.

Although no associations between circulating levels of CRP, IL-6 and TNF-α and risk of colorectal adenomas were found overall, several other issues should be considered when interpreting the results. Circulating CRP levels were associated with an increased risk of multiple small tubular or advanced colorectal adenomas, and the association was more pronounced among current smokers and never/former nonsteroidal anti-inflammatory drugs users [10]. Although circulating levels of CRP was not associated with risk of small colorectal adenomas (<5 mm), a positive association was found with risk of large colorectal adenomas (≥5 mm) [22]. In addition, high levels of circulating CRP (>2.95 mg/L) was inversely associated with risk of tubular adenomas in the CLUE II cohort [23]. A positive association was also found between TNF-α and risk of tubular adenomas [12]. Circulating IL-6 levels was found associated with the risk of colorectal adenomas among subjects with homeostasis model assessment of insulin resistance≥1.73, but not among those<1.73 [15]. However, the limited information precluded a more robust assessment of the above-mentioned findings. The pooled results on CRP and IL-6 and risk of colorectal adenomas are consistent with those from nested case-control studies [8, 17, 23] in which circulating levels measurement preceded colorectal adenomas incidence. No nested case-control study on circulating levels of TNF-α and colorectal adenomas was identified.

Other limitations should also be of concern. First, serum levels were only measured once, thus the results may not precisely reflect an individual's true or long-term levels. However, the previous study showed that CRP levels were relatively stable over a 5-year period [17]. Although adipokine tissue concentrations may be more relevant to adenoma risk, circulating measurements are more useful in clinical settings. Second, serum levels of cytokines were measured after the diagnosis of adenoma, thus the possibility of reverse causality should be considered. However, colorectal adenomas unlikely affect the amount of adipose tissue that is the major source of adipokines. Third, the confounding of smoking, body mass index and nonsteroidal anti-inflammatory drugs, which are related to both colorectal adenomas and adipokines, was not assessment because of the limited data available. Finally, only part of the studies was included in dose-response analysis, and publication bias should also be interpreted cautiously because of the relatively small number of studies.

Discovery of reliable biomarkers for assessment of colorectal neoplasia risk in a screening-aged population has presented challenges to date. Although inflammatory biomarkers have been speculated to be possible candidate biomarkers for colorectal neoplasia, no associations were found between circulating levels of CRP, IL-6 and TNF-α and risk of colorectal adenomas in this meta-analysis. However, exploring the associations of other inflammatory biomarkers with risk of colorectal adenomas may provide novel clinical findings. Among the very sparse studies, macrophage inhibitory cytokine-1 was found meaningful for detection of advanced colorectal adenoma in the Nurses' Health Study [8]. The biological effects of CRP, IL-6 and TNF-α suggest that these inflammatory biomarkers are not specifically correlated with tissue-specific inflammation that is most relevant for colorectal neoplasia [5], thus multiplex arrays that can measure multiple biomarkers rather than a single biomarker may better evaluate the complex and dynamic nature of inflammatory responses in future clinical research [5]. As for colorectal cancer, previous meta-analysis found that while C-reactive protein and interleukin-6 may be associated with risk of colorectal cancer [38–39], the few studies on tumor necrosis factor-α and risk of colorectal cancer mainly found null results [40–41]. Given the inconsistent results among publications available, further collaborative consortia and Mendelian randomization studies are still encouraged before inflammatory markers, especially C-reactive protein, are intended as screening tool for patients with increased risk of colorectal cancer [42].

In summary, circulating levels of CRP, IL-6 and TNF-α may be not useful biomarkers for identifying colorectal adenomas. Further prospective cohort studies are warranted to confirm these findings.

## MATERIALS AND METHODS

### Literature search and selection

We performed a literature search from inception to February 2016 using the databases of PubMed, Embase and Medline. Details of the search strategy are shown in Appendix 2. Moreover, we also reviewed the reference lists from retrieved articles to search for further relevant studies. There is no protocol for this meta-analysis.

Two investigators (ZXQ and LSL) independently reviewed all identified studies, and studies were included if they met the following criteria: (i) an observational study published as an original study; (ii) the exposure of interest was circulating levels of CRP, IL-6 or TNF-α; (iii) the outcome of interest was colorectal adenomas (studies focusing on recurrence of colorectal adenomas were excluded) and (iv) relative risk or odds ratio (OR) with 95% confidence interval (CI) were provided (we presented all results with OR), or mean and standard deviation were available from which an OR could be calculated (http://www.campbellcollaboration.org/escalc/html/EffectSizeCalculator-SMD-main.php, accessed 4/212016). If data were duplicated in more than one study, we included the study with the most recent one; otherwise, the one with the most number of cases was included.

### Data extraction

We extracted all data using a standardized data collection form (ZXQ and LSL). Information was recorded as follows: the first author's last name, publication year, number of cases and participants, measurement of exposure, country where the study was performed, variables adjusted for in the analysis, OR estimates with corresponding 95% CI of colorectal adenomas. For dose–response analysis, the number of cases and participants (person-years) and OR (95% CI) for each category of CRP, IL-6 and TNF-α were also extracted. We extracted the ORs that reflected the greatest degree of control for potential confounders.

### Statistical analysis

Pooled measure was calculated as the inverse variance weighted mean of the logarithm of OR with 95% CI to assess the strength of association. OR (95% CI) was calculated with the mean and standard deviation if it was not provided. A random-effect model was used as the pooling method, which considers both within-study and between-study variation. The I2 was used to assess heterogeneity, and I2 values of 25, 50 and 75% represent low, moderate and high heterogeneity, respectively [43]. Publication bias was evaluated with Egger regression test. Meta-regression and subgroup analysis were conducted to explore potential sources of heterogeneity and perform comparison between groups, and the p values from meta-regression were calculated with a permutation test of 1000 to control the spurious findings [44]. When moderate or higher heterogeneity was found and the heterogeneity cannot be explained by meta-regression, sensitivity of between-study heterogeneity was conducted to assess the robustness of conclusions [45]. We also performed a sensitivity analysis in which one study at a time was removed and the rest were analyzed to evaluate whether the results could have been affected markedly by a single study. Study quality was assessed using the 9-star Newcastle-Ottawa Scale (http://www.ohri.ca/programs/clinical_epidemiology/oxford.asp, accessed 4/21/2016).

For dose–response analysis, a two-stage random-effects dose–response meta-analysis [46] was performed to compute the trend from the correlated log OR estimates taking into account the between-study heterogeneity. The median or mean level of circulating CRP, IL-6 and TNF-α for each category was assigned to the corresponding OR for every study. If the upper boundary of the highest category was not provided, we assumed that the boundary had the same amplitude as the adjacent category. In the first stage, a restricted cubic spline model with three knots at the 25th, 50th and 75th percentiles of the circulating levels was estimated using generalized least square regression taking into account the correlation within each set of published ORs. Then the study-specific estimates were combined using the restricted maximum likelihood method in a multivariate random-effects meta-analysis. A P value for nonlinearity was calculated by testing the null hypothesis that the coefficient of the second spline is equal to 0. All statistical analyses were performed with STATA version 12.0 (Stata Corporation, College Station, TX). All reported probabilities (P values) were two-sided with p<0.05 considered statistically significant.

## SUPPLEMENTARY MATERIALS APPENDIX, TABLES AND FIGURES






