# Supplementary material for: Circulating levels of C-reactive protein, interleukin-6 and tumor necrosis factor-α and risk of colorectal adenomas: a meta-analysis
Source: Oncotarget. 2016 Sep 6;7(39):64371–9. doi: 10.18632/oncotarget.11853 (PMC5325449; doi:10.18632/oncotarget.11853)
Supplement: Supplementary file 1 [file oncotarget-07-64371-s001.pdf]

Circulating levels of C-reactive protein, interleukin-6 and tumor necrosis factor-α and risk of colorectal adenomas: a meta-analysis

APPENDIX TABLES AND FIGURES

Appendix 1: PRISMA 2009 checklist

See Appendix File 1

Appendix 2: Search strategy

Search (Pubmed)	Query	Results in Pubmed	Results in Embase and Medline
#1	C-reactive protein	56,771	116,090
#2	CRP	33,347	61,194
#3	Interleukin-6	65,240	162,423
#4	IL-6	98,659	108,295
#5	tumor necrosis factor*	153,616	269,136
#6	TNF*	153,683	203,214
#7	colorectal	123,928	197,089
#8	colon	176,992	318,533
#9	rectal	98,711	118,864
#10	Neoplas*	2,391,690	952,140
#11	polyp*	169,616	339,423
#12	adenoma*	100,288	117,686
#13	#1 OR #2 OR #3 OR #4 OR #5 OR #6	286,379	507,830
#14	#7 OR #8 OR #9	334,972	526,386
#15	#10 OR #11 OR #12	2,557,943	1,338,980
#16	#13 AND #14 AND #15	2,957	3,367

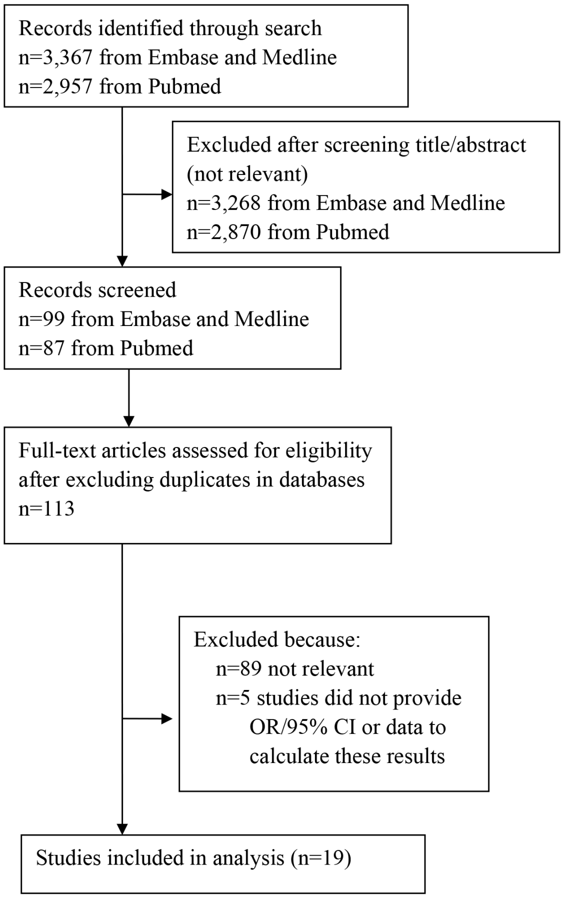

Appendix 3: Characteristics of studies on CRP, IL-6 and TNF-α included in the meta-analysis

See Appendix File 3

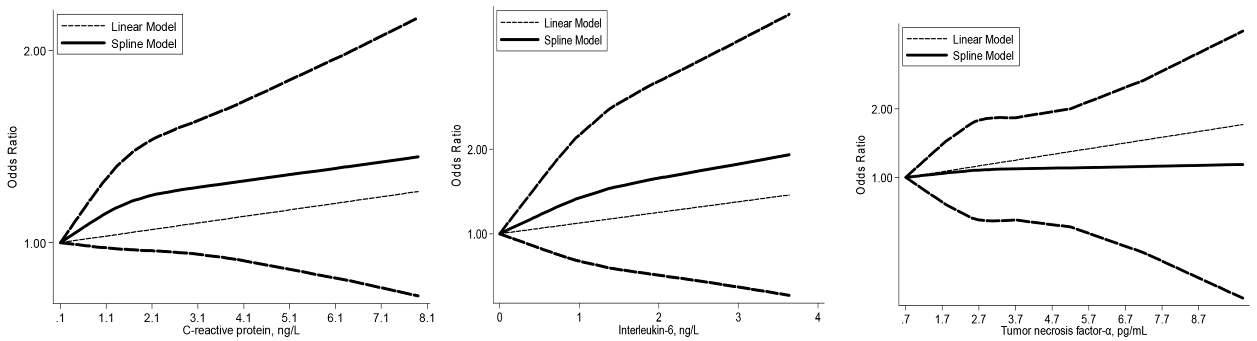

Appendix 4: Dose-resposne analysis. The dose–response analysis between circulating levels of C-reactive protein, interleukin-6 and tumor necrosis factor-α and risk of colorectal adenomas. The solid and long dash lines represent the estimated odds ratio and 95% confidence intervals. The short dash lines represent the linear relation. The vertical axis is on a log scale. We used the lowest value in the included studies as the reference level.
