# Supplementary material for: Circulating levels of C-reactive protein, interleukin-6 and tumor necrosis factor-α and risk of colorectal adenomas: a meta-analysis
Source: Oncotarget. 2016 Sep 6;7(39):64371–9. doi: 10.18632/oncotarget.11853 (PMC5325449; doi:10.18632/oncotarget.11853)
Supplement: Supplementary file 3 [file oncotarget-07-64371-s003.doc]

Characteristics of studies on CRP, IL-6 and TNF-α included in the meta-analysis

| Author, Year, [ref.] | Cases/  Controls  (N) | Mean age (years) | Male (%) | Exposure | Assessment  method | Covariates adjusted for |
| --- | --- | --- | --- | --- | --- | --- |
| Inflammatory cytokines |  |  |  |  |  |  |
| Groblewska et al. 2008^1^, Poland, case-control | 38  35 | 55  44.5 | 65.8  42.9 | IL-6  CRP | ELISA  Immunoturbidimetric kits | --- |
| Kim et al. 2008^2^, USA, case-control | 242  631 | 58  54 | 59.5  39.6 | CRP (n/N)  IL-6  TNF-α | ELISA | Age, sex and obesity (BMI) |
| Park et al. 2008^3^, Korea, case-control | 1982  3505 | 62.3  54.7 | 70.8  59.1 | CRP | Immuno-nephelometry | Age, sex, BMI, history of medication aspirin, postmenopausal hormones, multivitamins), fasting glucose, smoking and family history of colorectal cancer |
| Tsilidis et al. 2008^4^, USA, NCC, CLUE II cohort | 135  269 | 55.3  55.1 | 51.1  51.3 | CRP | A high sensitivity assay | Age, sex, race, date of blood draw, time since last meal, type of endoscopy, cigarette smoking status, BMI at baseline, BMI at age 21, ever use of estrogen or progesterone (women), use of aspirin or non-steroidal anti-inflammatory drugs, use of diabetes medications, family history of colorectal cancer |
| Otake et al. 2009^5^, Japan, case-control | 646  635 | 52.0  51.8 | 100  100 | CRP | Immunonephelometric assay | Plasma kept frozen at-80^o^C  Age, hospital, plasma/serum status, rank in the Self Defense Forces, cigarette smoking, alcohol use, BMI, physical activity, and parental colorectal cancer |
| Kang et al. 2010^6^, Korea, MCC | 1122  1122 | 56.0  56.0 | 77.2  77.2 | CRP | Automatic analyzer | --- |
| Ognjanovic et al. 2010^7^, USA, MCC | 271  539 | 62.5  62.0 | 67.9  68.8 | IL-6  CRP | A high-sensitivity  ELISA assay  A high-sensitivity  turbidity assay | Sex, age, race, screening date, recruitment clinic, smoking status and BMI |
| Otake et al. 2010^8^, Japan, case-control | 47  26 | 65.1  67.9 | 100  100 | CRP | Nephelometry | --- |
| Yamaji et al. 2010^9^, Japan, MCC | M  523  480  F  255  255 | 69.5  69.5  59.5  59.5 | 100  100  0  0 | TNF-α | Human Serum Adipokine (Panel B) LINCOplexKit | Age, screening period, duration of fasting, BMI, cigarette smoking, alcohol drinking, family history of colorectal cancer, and nonsteroidal anti-inflammatory drug use |
| Gunter et al. 2011^10^, USA, NCC, PLCO trial | 356  396 | 62.7  62.8 | 65.2  65.4 | CRP | solid phase chemiluminescent immunometric assay | Age, gender, fiscal year at study entry, race, screening center, study protocol, and season of blood draw, cigarette smoking status, BMI at baseline, use of NSAIDs, diabetes, use of hormone therapy (females only), family history of colorectal cancer, and educational attainment |
| Hosono et al. 2012^11^, Japan, MCC | 62  34 | 67.7  67.6 | 62.9  58.8 | TNF-α | ELISA | Age |
| Sasaki et al. 2012^12^, Japan, MCC | 118  218 | 52  51 | 100  100 | IL-6 | ELISA | Age, current smoking, alcohol consumption, family history of CRC, BMI, HOMA-IR and insulin |
| Kang et al. 2013^13^, USA  Case-control | 138  324 | 57.0  55.2 | 55  47 | TNF-α  IL-6 | ELISA | --- |
| Vaughn et al. 2013^14^, USA, case-control | 401  1050 | 57.5  54.6 | 46.6  33.4 | IL-6  TNF-α | ELISA  Panel B method | Age, sex, race, non-steroidal anti-inflammatory use, BMI, family history of colorectal cancer, and smoking status |
| Comstock et al. 2014^15^, USA, case-control | 37  69 | 57  57 | 100  100 | TNF-α | Human Cytokine/  Chemokine panel | Age and smoking |
| Kimura et al. 2014^16^, Japan, case-control | 41  40 | 64.1  56.0 | 58.5  55.0 | TNF-α |  | --- |
| Davenport et al. 2015^17^, USA, MCC | 226  226  198  198  283  283 | 57.5  56.6  60.0  58.9  58.9  57.4 | 72.6  72.6  79.3  79.3  71.7  71.7 | CRP | High Sensitivity Wide  Range Kit | Age, sex, race, educational attainment, and study site |
| Henry et al. 2015^18^, USA, case-control | 97  97 | 66.8  63.2 | 60.2  39.8 | IL-6  TNF-α | Human Cytokine/ Chemokine Magnetic Bead Panel Immunoassay | Age, sex, and previous screening |
| Song et al. 2016^19^, USA, NCC, Nurses' Health Study^19^ | 757  757 | 56.9  56.8 | 0  0 | CRP  IL-6 | a highly  sensitive immunoturbidimetric assay  ELISA | Age, date of endoscopy, birth year, indication for endoscopy, time period of any prior endoscopy, month and year of blood draw, fasting status, family history of colorectal cancer, multivitamin use, pack-years of smoking before age 30, alcohol consumption, BMI, physical activity, regular aspirin/NSAID use, postmenopausal status and hormone  use, calcium intake, Alternative Healthy Eating Index, and other plasma markers (MIC-1, sTNFR-2, CRP for IL-6; MIC-1, sTNFR-2, IL-6 for CRP) |

**References**

1. Groblewska M, Mroczko B, Wereszczynska-Siemiatkowska U, Kedra B, Lukaszewicz M, Baniukiewicz A, et al. Serum interleukin 6 (IL-6) and C-reactive protein (CRP) levels in colorectal adenoma and cancer patients. Clin Chem Lab Med 2008;46:1423-1428.

2. Kim S, Keku TO, Martin C, Galanko J, Woosley JT, Schroeder JC, et al. Circulating levels of inflammatory cytokines and risk of colorectal adenomas. Cancer Res 2008;68:323-328.

3. Park SK, Park DI, Park JH, Kim HJ, Cho YK, Sohn CI, et al. [C-reactive protein level and colorectal adenoma]. Korean J Gastroenterol 2008;51:225-231.

4. Tsilidis KK, Erlinger TP, Rifai N, Hoffman S, Hoffman-Bolton J, Helzlsouer KJ, et al. C-reactive protein and colorectal adenoma in the CLUE II cohort. Cancer Causes Control 2008;19:559-567.

5. Otake T, Uezono K, Takahashi R, Fukumoto J, Tabata S, Abe H, et al. C-reactive protein and colorectal adenomas: Self Defense Forces Health Study. Cancer Sci 2009;100:709-714.

6. Kang HW, Kim D, Kim HJ, Kim CH, Kim YS, Park MJ, et al. Visceral obesity and insulin resistance as risk factors for colorectal adenoma: a cross-sectional, case-control study. Am J Gastroenterol 2010;105:178-187.

7. Ognjanovic S, Yamamoto J, Saltzman B, Franke A, Ognjanovic M, Yokochi L, et al. Serum CRP and IL-6, genetic variants and risk of colorectal adenoma in a multiethnic population. Cancer Causes Control 2010;21:1131-1138.

8. Otake S, Takeda H, Fujishima S, Fukui T, Orii T, Sato T, et al. Decreased levels of plasma adiponectin associated with increased risk of colorectal cancer. World J Gastroenterol 2010;16:1252-1257.

9. Yamaji T, Iwasaki M, Sasazuki S, Tsugane S. Interaction between adiponectin and leptin influences the risk of colorectal adenoma. Cancer Res 2010;70:5430-5437.

10. Gunter MJ, Cross AJ, Huang WY, Stanczyk FZ, Purdue M, Xue X, et al. A prospective evaluation of C-reactive protein levels and colorectal adenoma development. Cancer Epidemiol Biomarkers Prev 2011;20:537-544.

11. Hosono K, Yamada E, Endo H, Takahashi H, Inamori M, Hippo Y, et al. Increased tumor necrosis factor receptor 1 expression in human colorectal adenomas. World J Gastroenterol 2012;18:5360-5368.

12. Sasaki Y, Takeda H, Sato T, Orii T, Nishise S, Nagino K, et al. Serum Interleukin-6, insulin, and HOMA-IR in male individuals with colorectal adenoma. Clin Cancer Res 2012;18:392-399.

13. Kang M, Edmundson P, Araujo-Perez F, McCoy AN, Galanko J, Keku TO. Association of plasma endotoxin, inflammatory cytokines and risk of colorectal adenomas. BMC Cancer 2013;13:91.

14. Vaughn CB, Ochs-Balcom HM, Nie J, Chen Z, Thompson CL, Tracy R, et al. No Association between Circulating Levels and Genetic Variants of IL-6 and TNF-alpha and Colon Adenoma. Gastroenterology Res 2013;6.

15. Comstock SS, Hortos K, Kovan B, McCaskey S, Pathak DR, Fenton JI. Adipokines and obesity are associated with colorectal polyps in adult males: a cross-sectional study. PLoS One 2014;9:e85939.

16. Kimura Y, Matsumoto H, Oosawa M, Fujita M, Tarumi K, Kamada T, et al. [Relationship between visceral fat and development of colorectal neoplasms using computed tomographic colonography and adipocytokine levels]. Nihon Shokakibyo Gakkai Zasshi 2014;111:2121-2130.

17. Davenport JR, Cai Q, Ness RM, Milne G, Zhao Z, Smalley WE, et al. Evaluation of pro-inflammatory markers plasma C-reactive protein and urinary prostaglandin-E2 metabolite in colorectal adenoma risk. Mol Carcinog 2015.

18. Henry CJ, Sedjo RL, Rozhok A, Salstrom J, Ahnen D, Levin TR, et al. Lack of significant association between serum inflammatory cytokine profiles and the presence of colorectal adenoma. BMC Cancer 2015;15:123.

19. Song M, Mehta RS, Wu K, Fuchs CS, Ogino S, Giovannucci EL, et al. Plasma Inflammatory Markers and Risk of Advanced Colorectal Adenoma in Women. Cancer Prev Res (Phila) 2016;9:27-34.
